# Supplementary material for: Algal symbiont diversity in Acropora muricata from the extreme reef of Bouraké associated with resistance to coral bleaching
Source: PLoS One. 2024 Feb 28;19(2):e0296902. doi: 10.1371/journal.pone.0296902 (PMC10901360; doi:10.1371/journal.pone.0296902)
Supplement: S1 Table — Bouraké healthy (BZ), Bouraké bleached (BB), and reference healthy (RZ) in May 2021 (T2) according to their initial category and origin. (DOCX) [file pone.0296902.s001.docx]

**S1 Table**. **Mortality (%) of tagged colonies of Acropora muricata.** Bouraké healthy (BZ), Bouraké bleached (BB), and reference healthy (RZ) in May 2021 (T2) according to their initial category and origin.

| **Colony # BZ BB RZ** |
| --- |
| 1 10 100 lost |
| 2 0 100 lost |
| 3 100 100 lost |
| 4 25 100 0 |
| 5 15 100 0 |
| 6 0 50 0 |
| 7 40 30 0 |
| 8 5 100 0 |
| 9 40 100 0 |
| 10 100 100 |
| 11 100 100 |
| 12 0 100 |
| 13 0 20 |
| 14 30 100 |
| 15 0 100 |
