# Supplementary material for: Algal symbiont diversity in Acropora muricata from the extreme reef of Bouraké associated with resistance to coral bleaching
Source: PLoS One. 2024 Feb 28;19(2):e0296902. doi: 10.1371/journal.pone.0296902 (PMC10901360; doi:10.1371/journal.pone.0296902)
Supplement: S2 Table — T-test on one-way PERMANOVA for each coral physiological trait measured in Acropora muricata during the bleaching (T1) and post-bleaching (T2). (DOCX) [file pone.0296902.s002.docx]

**S2 Table**. **Pairwise comparison on coral physiological traits**. T-test on one-way PERMANOVA for each coral physiological trait measured in Acropora muricata during the bleaching (T1) and post-bleaching (T2).

| **T1 (Bleaching)** |
| --- |
| **Trait Comparison T p Unique perms** |
| P_gross_ BB *vs* BZ 8.841 **<0.001** 9934 |
| BB *vs* RZ 6.639 **<0.001** 9908 |
| BZ *vs* RZ 1.671 0.102 987 |
| Dark respiration BB *vs* BZ 3.932 **<0.001** 9915 |
| RZ *vs* BB 2.566 0.011 9897 |
| BZ vs RZ 1.141 0.164 9854 |
| P_g_:R BB *vs* BZ 6.002 **<0.001** 9941 |
| BB *vs* RZ 4.971 **<0.001** 9876 |
| BZ *vs* RZ 0.125 0.902 9868 |
| Symbio. BB *vs* BZ 20.48 **<0.001** 1712 |
| BB *vs* RZ 18.96 **<0.001** 1714 |
| BZ *vs* RZ 3.810 **0.004** 1710 |
| Chlorophyll_tot_ BB *vs* BZ 4.763 **<0.001** 1704 |
| BB *vs* RZ 4.687 **0.002** 1704 |
| BZ *vs* RZ 1.975 **0.064** 1695 |
| Yield_max_ BB *vs* BZ 4.189 **<0.001** 9893 |
| BB *vs* RZ 0.118 0.-03 9141 |
| BZ *vs* RZ 4.359 **<0.001** 9863 |
| rETR_max_ BB *vs* BZ 5.348 **<0.001** 9926 |
| BB *vs* RZ 2.446 **0.026** 9630 |
| BZ *vs* RZ 3.76 **0.001** 9895 |
| Carbohydrates BB *vs* BZ 4.073 **<0.001** 9942 |
| BB *vs* RZ 0.491 0.628 9545 |
| BZ *vs* RZ 3.421 **0.002** 9872 |
| Lipids BB *vs* BZ 2.658 **0.012** 9839 |
| BB *vs* RZ 1.398 0.176 9805 |
| BZ *vs* RZ 0.9306 0.366 9805 |
| Calcification BB *vs* BZ 5.418 <**0.001** 815 |
| BB *vs* RZ 7.043 **0.011** 84 |
| BZ *vs* RZ 2.625 **0.017** 9065 |
| **T2 (Post-bleaching)** |
| **Trait Comparison T p Unique perms** |
| P_gross_:R BB *vs* BZ 0.693 0.485 7668 |
| BB *vs* RZ 1.852 0.125 84 |
| BZ *vs* RZ 2.858 **0.012** 7668 |
| Symbio. BB *vs* BZ 0.572 **0.554** 394 |
| BB *vs* RZ 2.083 0.712 84 |
| BZ *vs* RZ 4.570 **<0.001** 6813 |
| Proteins BB *vs* BZ 0.152 0.895 455 |

| **Table S2 (continued)** |
| --- |
| **T2 (Post-bleaching)** |
| **Trait Comparison T p Unique perms** |
| BB *vs* RZ 1.952 **0.040** 84 |
| BZ *vs* RZ 3.329 **0.006** 7663 |
| Carbohydrates BB *vs* BZ 0.572 0.572 455 |
| BB *vs* RZ 5.148 **0.012** 84 |
| BZ *vs* RZ 4.437 **0.002** 7640 |
| Biomass BB *vs* BZ 1.163 0.270 455 |
| BB *vs* RZ 0.87 0.507 84 |
| BZ *vs* RZ 2.902 **0.005** 7640 |
