# Supplementary material for: Algal symbiont diversity in Acropora muricata from the extreme reef of Bouraké associated with resistance to coral bleaching
Source: PLoS One. 2024 Feb 28;19(2):e0296902. doi: 10.1371/journal.pone.0296902 (PMC10901360; doi:10.1371/journal.pone.0296902)
Supplement: S3 Table — T-test on one-way PERMANOVA for each metabolic reserve (i.e., proteins, lipids, carbohydrates, and biomass) measured in Acropora muricata post-bleaching (T2). Only the colonies that survived at T2 were considered. (DOCX) [file pone.0296902.s003.docx]

**S3 Table**. **Pairwise comparison on metabolic reserves.** T-test on one-way PERMANOVA for each metabolic reserve (i.e., proteins, lipids, carbohydrates, and biomass) measured in Acropora muricata post-bleaching (T2). Only the colonies that survived at T2 were considered.

**Reserves Category T p Unique perms**

Biomass BB 1.488 0.106 10

BZ 5.961 **<0.001** 9679

RZ 0.248 0.865 462

Lipids BB 2.117 0.203 10

BZ 6.755 **<0.001** 9713

RZ 3.191 **0.01** 462

Proteins BB 2.766 0.09 10

BZ 3.834 **0.005** 9715

RZ 1.03 0.349 462

Carbohydrates BB 0.212 0.899 10

BZ 1.902 0.065 9332

RZ 4.842 **0.004** 462
