# Supplementary figures and images for: Algal symbiont diversity in Acropora muricata from the extreme reef of Bouraké associated with resistance to coral bleaching
Source: PLoS One. 2024 Feb 28;19(2):e0296902. doi: 10.1371/journal.pone.0296902 (PMC10901360; doi:10.1371/journal.pone.0296902)

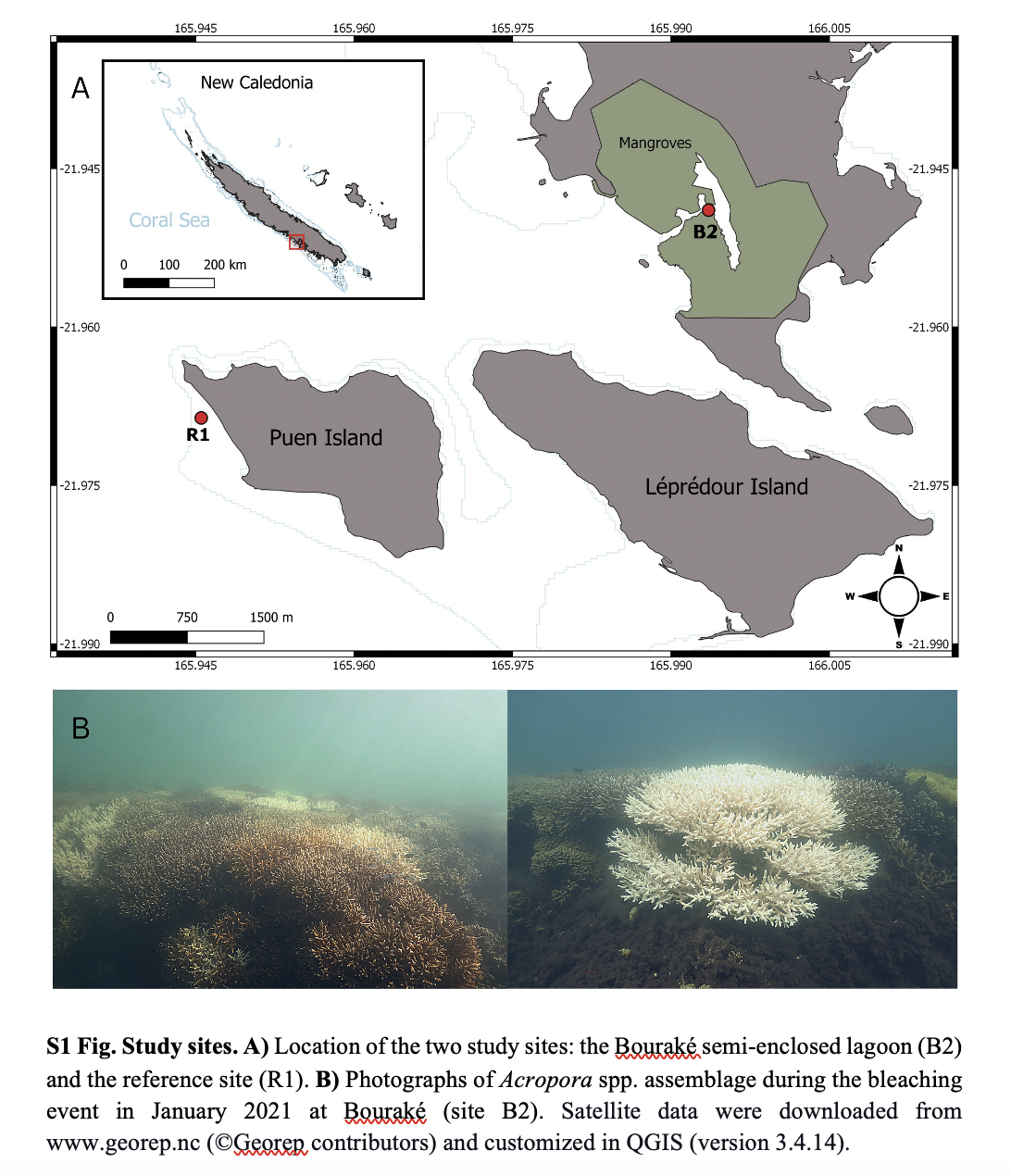

Supplement: S1 Fig — A) Location of the two study sites: the Bouraké semi-enclosed lagoon (B2) and the reference site (R1). B) Photographs of Acropora spp. assemblage during the bleaching event in January 2021 at Bouraké (site B2). Satellite data were downloaded from www.georep.nc (©Georep contributors) and customized in QGIS (version 3.4.14). (TIFF) [file pone.0296902.s004.tiff]

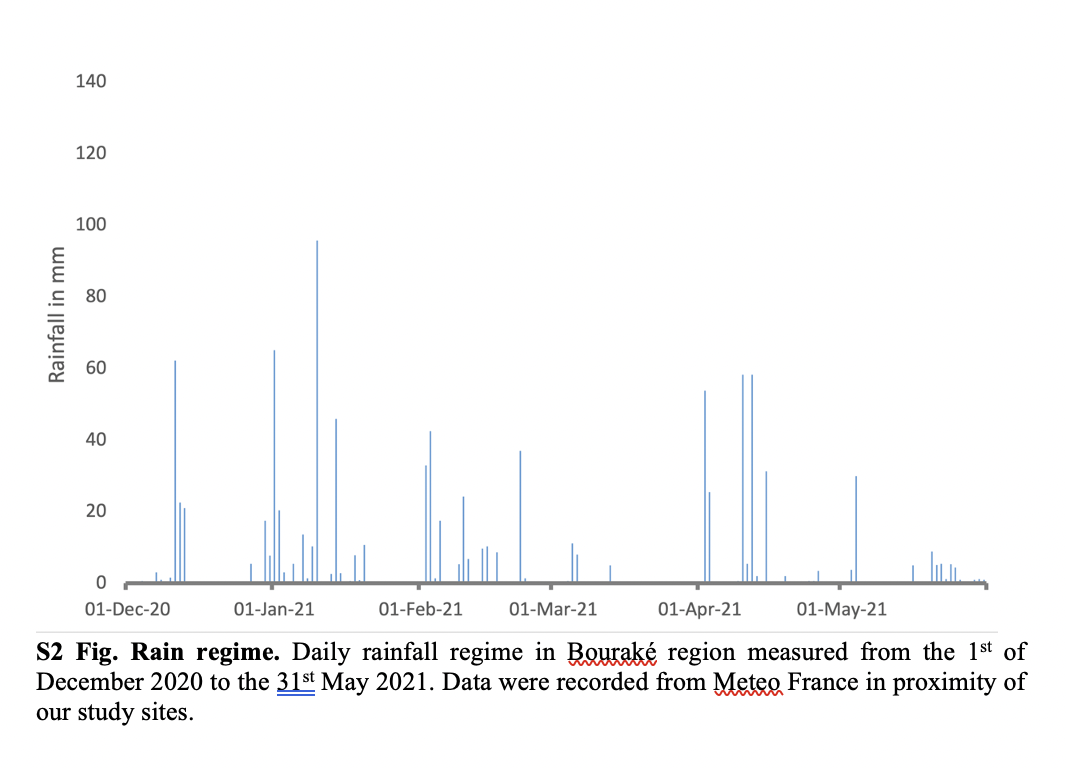

Supplement: S2 Fig — Daily rainfall regime in Bouraké region measured from the 1st of December 2020 to the 31st May 2021. Data were recorded from Meteo France in proximity of our study sites. (TIFF) [file pone.0296902.s005.tiff]

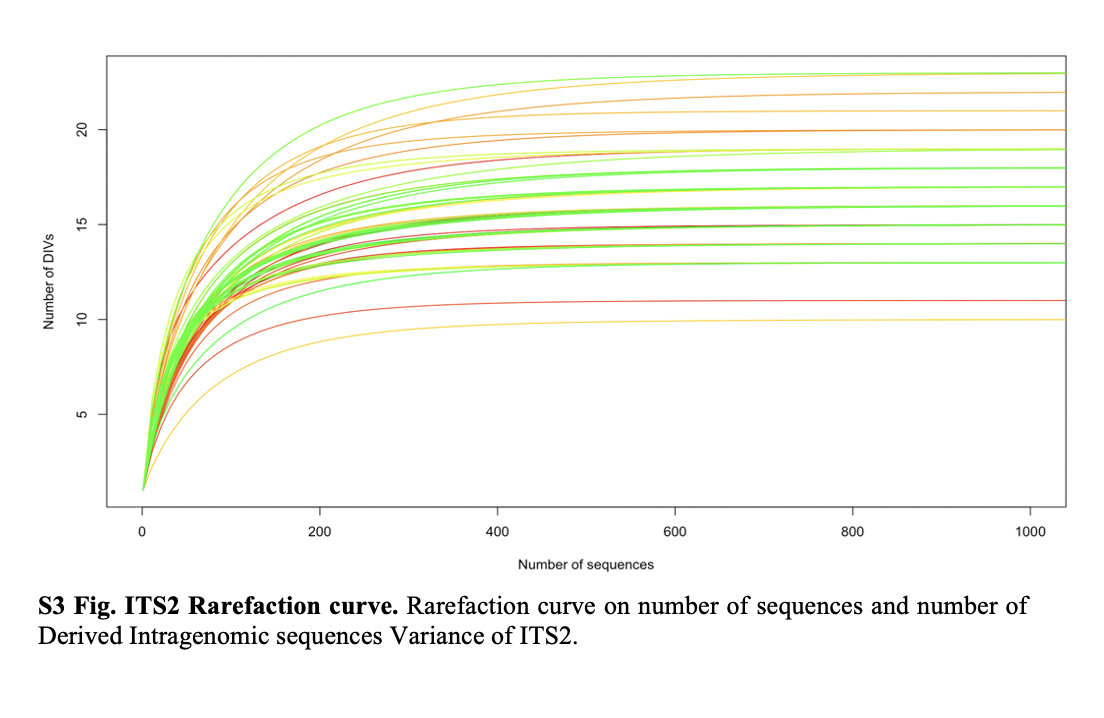

Supplement: S3 Fig — Rarefaction curve on number of sequences and number of Derived Intragenomic sequences Variance of ITS2. (TIFF) [file pone.0296902.s006.tiff]

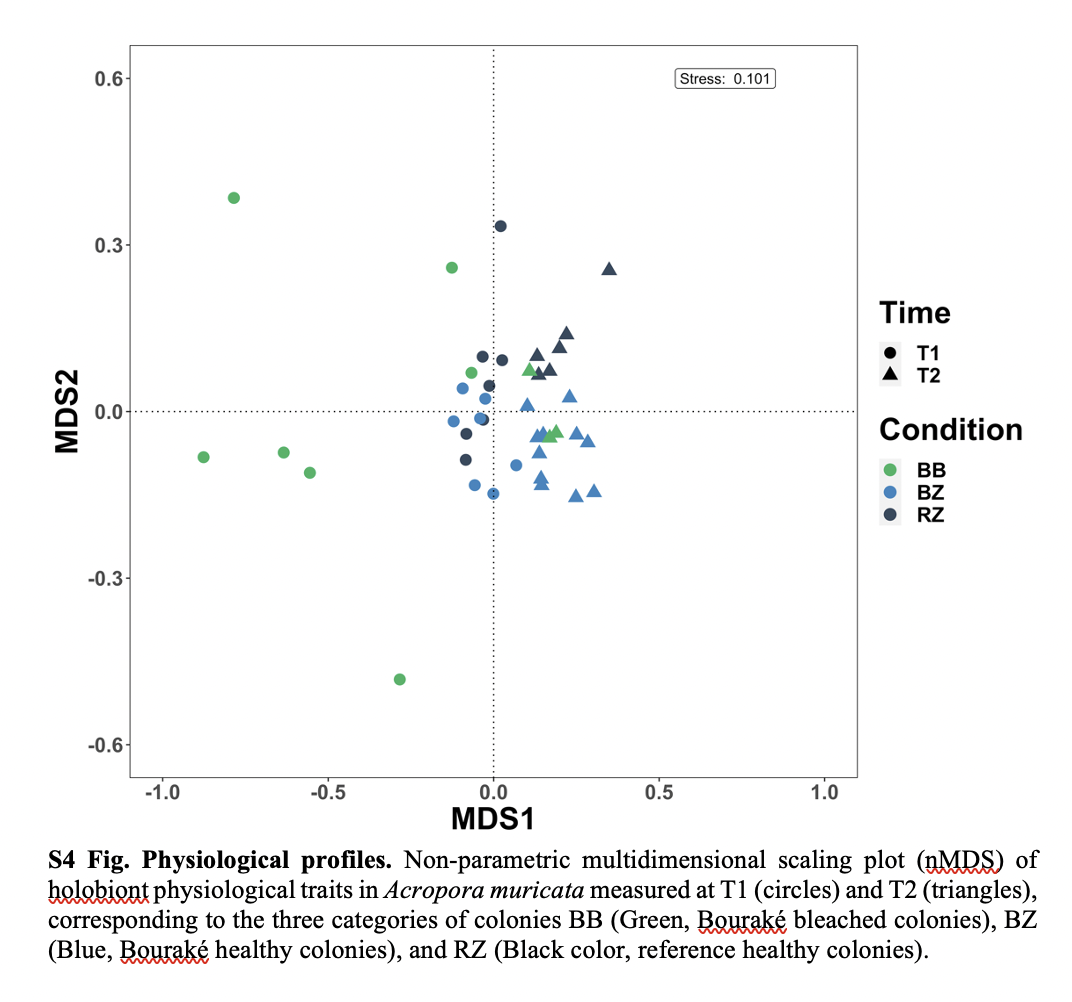

Supplement: S4 Fig — Non-parametric multidimensional scaling plot (nMDS) of holobiont physiological traits in Acropora muricata measured at T1 (circles) and T2 (triangles), corresponding to the three categories of colonies BB (Green, Bouraké bleached colonies), BZ (Blue, Bouraké healthy colonies), and RZ (Black color, reference healthy colonies). (TIFF) [file pone.0296902.s007.tiff]

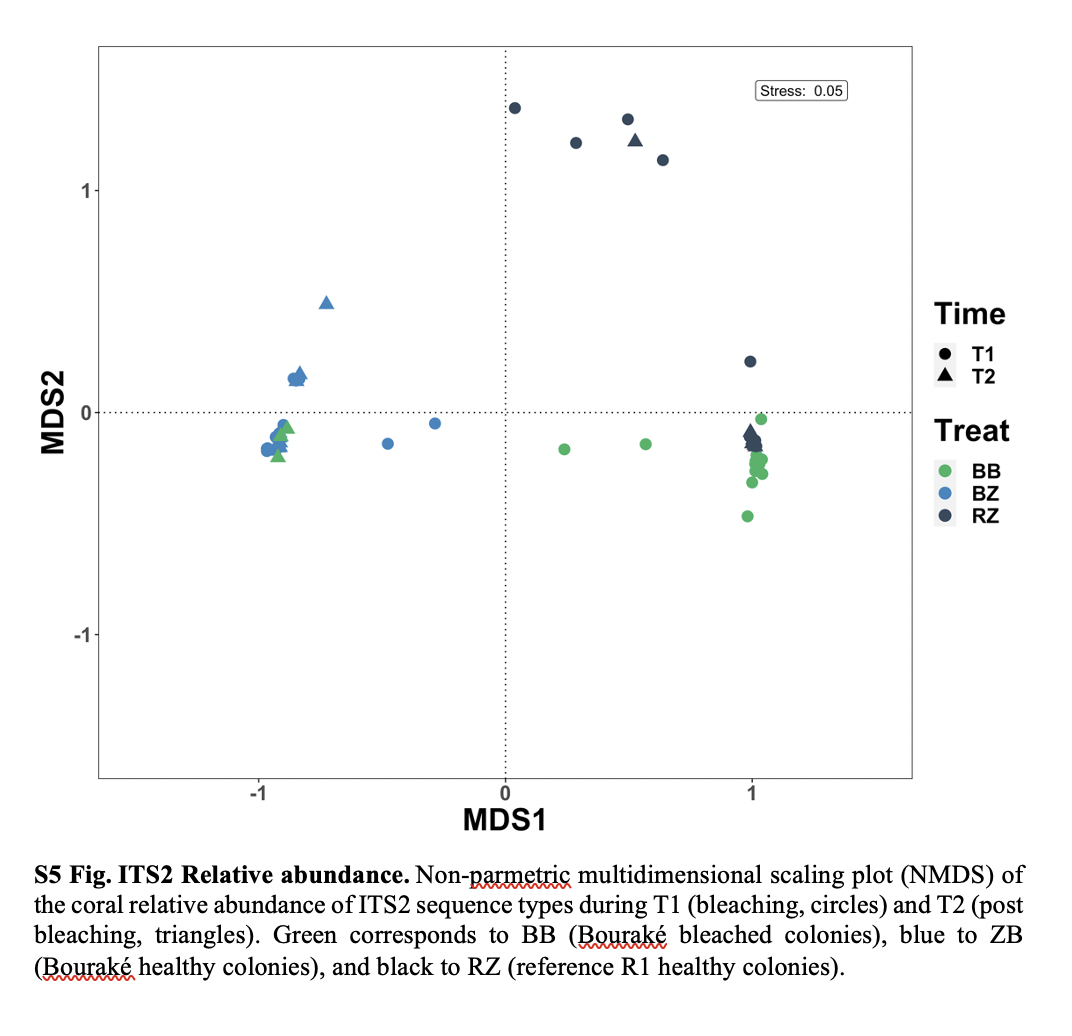

Supplement: S5 Fig — Non-parmetric multidimensional scaling plot (NMDS) of the coral relative abundance of ITS2 sequence types during T1 (bleaching, circles) and T2 (post bleaching, triangles). Green corresponds to BB (Bouraké bleached colonies), blue to ZB (Bouraké healthy colonies), and black to RZ (reference R1 healthy colonies). (TIFF) [file pone.0296902.s008.tiff]
