# Supplementary material for: Algal symbiont diversity in Acropora muricata from the extreme reef of Bouraké associated with resistance to coral bleaching
Source: PLoS One. 2024 Feb 28;19(2):e0296902. doi: 10.1371/journal.pone.0296902 (PMC10901360; doi:10.1371/journal.pone.0296902)
Supplement: Attachment — Submitted filename: review .pdf [file pone.0296902.s009.pdf]

PONE-D-23-04677

Intra-clade diversity provides resistance to coral bleaching under extreme environmental conditions in Acropora muricata

---

This study helps to improve our knowledge on the disparity of coral colony responses to marine heat waves, why within the same species, certain colonies resist better than others to heat stress? This manuscript provides crucial information allowing us to improve the forecasts about the future of coral reefs but also new useful knowledge to try to establish lines of resistant corals. In this manuscript, the results showing that the few bleached colonies that survived acquired the same ITS2 profiles of the unbleached resistant colonies are extremely interesting, they confirm the essential role of Symbiodiniaceae in resistance/resilience to heat stress and these results are essential to guide future protective measures. I also appreciated the honesty of the authors, who recognize that they cannot decipher if the bleaching was due to seawater warming or freshwater input and do not rule out any possibility.
The conclusions of this manuscript are important and innovative so I recommend it for publication, I only have minor corrections/suggestions listed below

Abstract
Cladocopium spp.

Introduction
L46 "Corals experiencing bleaching are particularly vulnerable since they lose up to 90% of the Symbiodiniaceae photosynthate carbon needed for the daily metabolic requirements (9)" Since the work of Muscatine, which now dates back to 1969!, other more recent works have been carried out , estimating in a more precise way the autotrophic C contribution to coral needs in case of bleaching e.g. Tremblay et al 2012 (in *Mar. Ecol. Prog. Ser.*)
L84 the authors compared healthy and bleached corals from Bouraké with healthy colonies from a reference site but did they observe also some bleached colonies at the reference site? (even if it is in lower proportions than in Bouraké)
L91 This sentence should be moved to the discussion section.

Methods
L95 Are light and UV data available for the different sites?
L124 The authors should add some data on the presence of this species in both Bouraké and reference sites (recovery percentage or others…). This way, it might be easier to consider the consequences on a larger scale of what has been observed here in this study.
L144 Wouldn't it be more appropriate to speak about growth rates instead of calcification rates when the buoyant weight technique is used? We usually consider that alkalinity anomaly technique and ^{45}Ca labelling allow to estimate the calcification rates
For the lipid measurements, is the reference 48 right?? [Folch et al (1956) A simple technique to rule out occlusion of right coronary artery after aortic valve surgery. Biol. Chem. 226,497-509]

Discussion
The discussion is clear and well written, the introductory paragraph summarizes the main findings of the paper well.

The observed changes in ITS2 are well related to what has been observed in previous works (Camp et al) and are completely consistent with what has been obtained previously . The results show for the first time remarkably the ability of ITS2 sequences (C1, C1b, C1h) to allow a better resistance/resilience to heat stress.
L482 the reference of Reynaud et al (2003) Interacting effects of CO2 partial pressure and temperature on photosynthesis and calcification in a scleractinian corals might be helpful for this part of the discussion
L540 “Heterotrophy has already been suggested to be a potential advantage for Bouraké’s corals due to the high loading of organic carbon content released” do we have any idea of the fluctuations of organic matter inside Bouraké through the year?

Again the conclusion makes a very nice recap of the main findings
Just on comment L559 what do the authors mean by “extreme corals” the notion is not clear
